# Macrophage Migration Inhibitory Factor Deficiency Ameliorates High-Fat Diet Induced Insulin Resistance in Mice with Reduced Adipose Inflammation and Hepatic Steatosis

**DOI:** 10.1371/journal.pone.0113369

**Published:** 2014-11-20

**Authors:** Orla M. Finucane, Clare M. Reynolds, Fiona C. McGillicuddy, Karen A. Harford, Martine Morrison, John Baugh, Helen M. Roche

**Affiliations:** 1 Institute of Molecular Medicine, School of Medicine, Trinity Centre for Health Sciences, St. James Hospital, Dublin 8, Ireland; 2 Nutrigenomics Research Group, School of Public Health & Population Science, UCD Conway Institute, University College Dublin, Dublin 4, Ireland; 3 Liggins Institute, University of Auckland, Auckland, New Zealand; 4 School of Medicine and Medical Science, UCD Conway Institute, University College Dublin, Dublin 4, Ireland; College of Tropical Agriculture and Human Resources, University of Hawaii, United States of America

## Abstract

Macrophage infiltration is a critical determinant of high-fat diet induced adipose tissue inflammation and insulin resistance. The precise mechanisms underpinning the initiation of macrophage recruitment and activation are unclear. Macrophage migration inhibitory factor (MIF), a pro-inflammatory cytokine, displays chemokine-like properties. Circulating MIF levels are elevated during obesity however its role in high-fat diet induced adipose inflammation and insulin resistance remains elusive. Wildtype and MIF^−/−^ C57Bl\6J mice were fed chow or high-fat diet. Body weight and food intake was assessed. Glucose homeostasis was monitored by glucose and insulin tolerance tests. Adipose tissue macrophage recruitment and adipose tissue insulin sensitivity was evaluated. Cytokine secretion from stromal vascular fraction, adipose explants and bone marrow macrophages was measured. Inflammatory signature and insulin sensitivity of 3T3-L1-adipocytes co-cultured with wildtype and MIF^−/−^ macrophage was quantified. Hepatic triacylglyceride levels were assessed. MIF^−/−^ exhibited reduced weight gain. Age and weight-matched obese MIF^−/−^ mice exhibited improved glucose homeostasis coincident with reduced adipose tissue M1 macrophage infiltration. Obese MIF^−/−^ stromal vascular fraction secreted less TNFα and greater IL-10 compared to wildtype. Activation of JNK was impaired in obese MIF^−/−^adipose, concomitant with pAKT expression. 3T3-L1-adipocytes cultured with MIF^−/−^ macrophages had reduced pro-inflammatory cytokine secretion and improved insulin sensitivity, effects which were also attained with MIF inhibitor ISO-1. MIF^−/−^ liver exhibited reduced hepatic triacyglyceride accumulation, enhanced pAKT expression and reduced NFκB activation. MIF deficiency partially protects from high-fat diet induced insulin resistance by attenuating macrophage infiltration, ameliorating adipose inflammation, which improved adipocyte insulin resistance *ex vivo*. MIF represents a potential therapeutic target for treatment of high-fat diet induced insulin resistance.

## Introduction

Adipose tissue inflammation is central to the pathogenesis of obesity associated insulin resistance (IR), type 2 diabetes and hepatic steatosis [1]–[3]. High-fat diet (HFD)-induced adipose tissue expansion is accompanied by a progressive infiltration of macrophages [4], [5]. Resident macrophages present immense heterogeneity and are broadly classified as pro-inflammatory M1 and anti-inflammatory M2 [6], [7]. At the onset of obesity M2 macrophages acquire an M1 phenotype [8], [9]. Pro-inflammatory cytokines including TNFα [10] and IL-6 produced by adipose tissue macrophages (ATM) exacerbate local inflammation promoting IR via down-regulation of IRS-1 and GLUT-4 [2]. Furthermore abrogation of Jun NH2-terminal kinase (JNK), a critical inflammatory regulator, in hematopoietic-derived cells protects mice from obesity-induced inflammation and IR [11]. Indisputably ATM infiltration and subsequent local inflammation is paramount for induction of IR, however the signals responsible for triggering macrophage recruitment remain ambiguous. Emerging data has highlighted the significance of adipose tissue-derived chemokines in driving macrophage recruitment during obesity. Deletion of CC-chemokine ligand CCL2/monocyte chemoattractant protein-1 (MCP-1) or its receptor CCR2 attenuates ATM recruitment concurrent with improved adipose tissue inflammation and systemic insulin sensitivity *in vivo* [12]–[15]. Several reports have challenged these findings indicating macrophage recruitment may occur independently of MCP-1 [15]–[17]. Furthermore, the importance of C-X-C chemokine ligand (CXCL)-5 [18], IL-8 [19] and CX3CL1 [20] has become apparent in both human and rodent models. Macrophage migration inhibitory factor (MIF) represents another potential candidate.

MIF is a pleiotropic cytokine, ubiquitously expressed and paramount in regulating inflammatory responses [21], [22]. Clinical studies suggest a link between MIF and adiposity. Circulating plasma MIF and mononuclear cell MIF mRNA expression are associated with increasing BMI and fatty acid concentration, impaired glucose tolerance and T2D [23]–[25], whereas reducing body weight or metformin treatment decreases serum MIF levels [26], [27]. Human and murine adipocytes express and secrete MIF [24], [28], [29]. Moreover glucose and insulin regulate MIF expression in adipocytes [30]. Recently MIF was identified as a non-cognate ligand of CXC chemokine receptors CXCR2 and CXCR4 in macrophages and T-cells respectively [31]. More recently in an atherosclerotic mouse model (LDLR^−/−^MIF^−/−^) maintained on a standard chow diet, absence of MIF reduced monocyte adhesion, macrophage lesion content, and atherosclerotic lesion size; coincident with improved glucose homeostasis [32].

Our study was undertaken to further define the impact of the obesogenic environment induced by high-saturated fat feeding on insulin sensitivity in a MIF-deficient setting. We hypothesized that lack of MIF protein would protect mice from the adverse effects of high-fat feeding by reducing ATM recruitment and improving adipose tissue immunophenotype. Additionally we examined the functional consequences of MIF^−/−^ macrophages on adipocyte biology and explored whether inhibition of exogenous MIF could block macrophage-induced adipocyte IR *in vitro.* This study highlights MIF as a critical mediator of ATM recruitment and regulator of adipose tissue inflammation during HFD-induced obesity.

## Methods

### Ethics statement

Ethical approval was obtained from UCD Ethics Committee and mice were maintained according to European Union and Irish Department of Health regulations. Body weight was monitored prior to and after all metabolic procedures to ensure full recovery of the animals. Any animals found to be exhibiting symptoms of pain or distress during or after procedure were euthanized immediately.

### Materials

Deoxy-D-glucose 2-[1,2-^3^H(N)]-was purchased from Perkin-Elmer Analytical Sciences (Dublin, Ireland). Cell culture material was purchased from Lonza (Slough, UK). All other reagents unless otherwise stated were from Sigma Aldrich Ltd.

### Animals

C57BL/6J wildtype (WT) mice were purchased from Charles River, Ireland. C57BL/6J MIF^−/−^ mice were generous gift from Dr. Baugh and bred at University College Dublin (UCD) under pathogen free conditions. MIF^−/−^ mice were backcrossed for 10 generations onto C57BL/6J background. Male mice aged 8–9 weeks were fed HFD (45% kcal from palm oil, 20% kcal from protein, 35.1% kcal from carbohydrates) (Research Diets Inc., USA) or chow diet (17% kcal from fat, 25% kcal from protein, 58% kcal from carbohydrates) (Harlan Teklad UK) *ab libitum* for 16 weeks. Body weight and food intake were recorded weekly. Mice were deemed to be obese when they weighed greater than 35g. Lean and obese mice were overnight fasted and injected with NaCl (pH 5.0) or insulin (0.75 U insulin/kilogram (kg), bodyweight (bw); Actrapid, Novo Nordisk, Denmark). After 15 minutes, mice were killed and plasma and tissue harvested.

### Body mass composition

Body mass composition was measured using Bruker's minispec LF50 body composition analyzer (Bruker Optik GmbH, Germany). Lean tissue, fat and fluid was calculated based on body weight of mouse (University College Cork/Alimentary Pharmabiotic Centre).

### Glucose and insulin tolerance test (GTT/ITT)

GTT and ITTs were performed on 4–6 h fasted mice. Mice were intraperitoneally injected (i.p.) with 25% (w/v) glucose (1.5 g glucose/kg bw); Braun Medical Ltd, Dublin, Ireland) or insulin (0.75 U/kg bw) respectively. Blood glucose levels were measured prior to administration and 15, 30, 60, 90, 120 minutes post glucose/insulin challenge using Accu-Chek glucometer (Roche Ltd, Dublin, Ireland).

### Insulin secretory response

Overnight fasted mice were subjected to i.p. glucose challenge (1.5 g glucose/kg bw). Blood samples were collected by tail vein bleed prior to and 30, 60 minutes post glucose challenge. Plasma insulin secretory response was determined using an ultra-sensitive insulin ELISA kit (Crystal Chem Inc., USA).

### Isolation of stromal vascular fraction (SVF) and flow cytometry

To separate the SVF from adipocytes, epididymal adipose tissue (EAT) was minced then collagenase (2 mg/ml) digested for 70 minutes. Adipocytes were removed and digested EAT suspension was filtered and centrifuged for 5 minutes at 1,700 rpm. The SVF was re-suspended and blocked in 2% BSA/PBS. Cells were stained with fluorescently labelled antibodies; F4/80-FITC, CD11B-AF647/PE, CD11C-RPE, CD3-APC, CD4-FITC, CD8-PE (AbD Serotec, Kidlington, UK). Unstained, single stained and fluorescence minus one controls were used for setting compensation and gates. Flow cytometry was performed on Dako CyAn ADP platform and analyzed using Summit v4.3 software (Beckman-Coulter Ltd., UK). Adipocytes and remaining SVF were seeded at 1×10^6^cells/ml and cultured in serum rich media (DMEM, 10% FBS and 1% penicillin/streptomycin) for 24 h. Media was harvested for cytokine profiling. Cells were re-suspended in TRI Reagent® for gene expression analysis.

### *Ex vivo* adipose tissue culture

EAT explants harvested from lean and obese mice (50 mg/well) were cultured in serum rich media for 24 h. Tissue was homogenized in 1 ml radioimmunoprecipitation assay (RIPA) buffer using a tissue lyser (Qiagen, West Sussex, UK).

### Insulin-stimulated glucose uptake into adipose explants

Adipose explants (50 mg) were placed in PBS+0.2% BSA prior to stimulation ± insulin (100 nM) for 15 min. ^3^H-glucose (0.1 mM 2-deoxyglucose+0.5 µCi/ml ^3^H-deoxyglucose) was added for 45 min. Tissue was washed, lysed in RIPA buffer and homogenized using a tissue lyser (Qiagen, West Sussex, UK). Glucose uptake was measured by liquid scintillation counting. Fold increase in glucose uptake over basal was calculated for each individual mouse.

### Cell culture

Bone marrow derived cells were isolated from femurs and tibias of 8–11 week old lean WT and MIF^−/−^ mice. Cells were cultured in serum rich media supplemented with 30% L929 conditioned medium for 7 days at 37°C to differentiate to bone marrow macrophage (BMM). On day 7 of differentiation macrophages were treated ± lipopolysaccharide (LPS) (10 ng/ml) for 30 minutes; cells were harvested for protein or incubated with fresh media for 6–24 h to measure cytokine secretion. 3T3L1-fibroblasts (ECACC, Salisbury, UK) were exposed to adipogenic medium containing 10% FBS, 0.5 mM isobutylmetylxanthine, 1 µM dexamethasone and 10 µg/ml insulin to induce differentiation.

### Co-culture assay

WT and MIF^−/−^ BMM were seeded on 0.4 µm Corning transwell filters, LPS-stimulated for 30 minutes and washed with PBS. Fresh media was added; transwells were transferred onto 3T3-L1 adipocytes (day 7 differentiation) and incubated for 72 h. Media was collected for cytokine analysis and 3T3-L1 adipocytes were harvested for gene expression analysis. On an identically treated 3T3-L1 adipocytes culture plate insulin-stimulated ^3^H- glucose transport assay was conducted.

### Conditioned media experiments

J774.2 macrophages were grown in serum rich media in T75 tissue culture flasks at a density of 2×10^6^cells/ml. Media was collected after 24 h from 1) unstimulated J774.2 macrophages, 2) J774.2 macrophages treated with recombinant (r) MIF (100 ng/ml), 3) J774.2 macrophages treated with the commercially available MIF inhibitor (S,R)-3-(4-Hydroxyphenyl)-4,5-dihydro-5-isoxazole acetic acid, methyl ester (ISO-1) (50 µM) (Merck, Ireland) and 4) J774.2 macrophages pretreated with ISO-1 (50 µM) for 1 h followed by rMIF (100 ng/ml) treatment for 24 h. Mature 3T3-L1 adipocytes were subsequently exposed to the collected conditioned media (CM) for 72 h. Cells were lysed in RIPA buffer and insulin-stimulated ^3^H-glucose transport assay into adipocytes was conducted.

### Glucose transport assay

Glucose transport assay was performed on 3T3-L1 adipocytes co-cultured with WT or MIF^−/−^ BMM or incubated with CM for 72 h. Cells were incubated in serum free media (DMEM +1% penicillin/streptomycin) for 2–5 h followed by glucose free incubation (0.2%BSA/PBS). Cells were stimulated ±insulin (100 nM) then [^3^H] Deoxyglucose (5 µCi/ml) cold glucose (1 mM) was added. Cells were washed, lysed in RIPA buffer at 4°C. [^3^H] Deoxyglucose uptake was measured by liquid scintillation counting.

### Statistical analysis

Data are reported as mean ± SEM. For GTT/ITT studies with multiple time-points an ANOVA was used to test for differences in means between WT and MIF^−/−^ groups. When ANOVA was significant post-hoc Bonferroni corrected t-tests were applied. AUC analysis was performed on GTT and ITT curves using Graphpad Prism 5 software (GraphPad Software Inc., San Diego, CA) calculated using the trapezoidal method. For comparison of data between two groups at a single time-point unpaired t-tests were performed. Statistical significance is presented as *p<0.05, **p<0.01 and ***p<0.001 in all figures.

### General laboratory techniques

Detailed description of plasma, gene expression, immunohistochemistry and immunoblot analysis are supplied in File S1.

## Results

### MIF deficiency partially protects mice from HFD-induced obesity and insulin resistance

**Figure 1:**
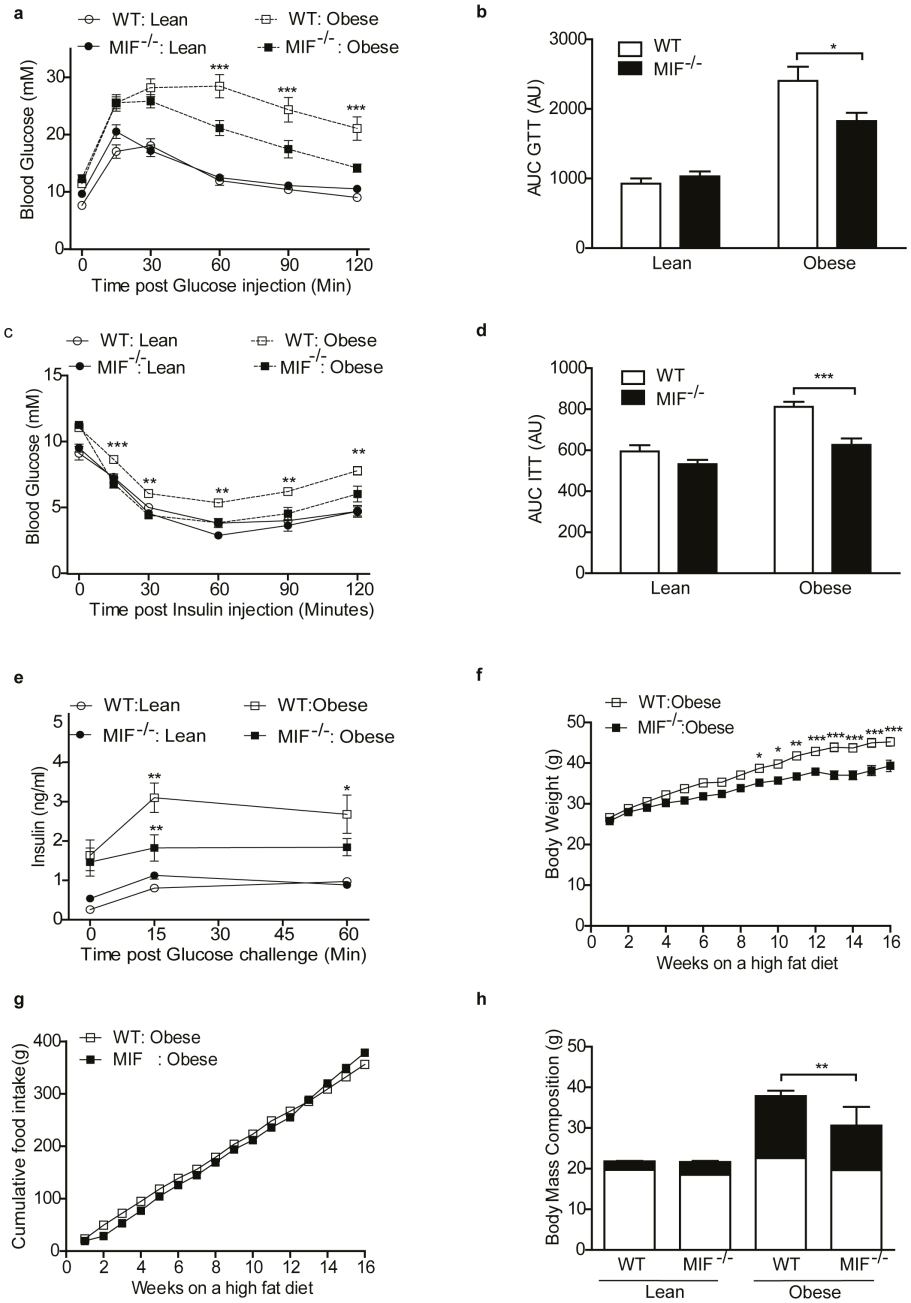
MIF deficiency partially protects from HFD-induced obesity and improves glucose homeostasis. (a) GTT (1.5 g glucose/kilogram (kg) body weight (BW)) in fasted lean and obese WT and MIF^−/−^ mice (white circles = WT lean; black circles =  MIF^−/−^ lean; white squares = WT obese; black squares = MIF^−/−^ obese). Lean n = 9, obese n = 9. (b) ITT (0.75 U insulin/kg BW) in fasted lean and obese WT and MIF^−/−^ mice (white circles = WT lean; black circles = MIF^−/−^ lean; white squares = WT obese; black squares = MIF^−/−^ obese). Lean n = 18, obese n = 18–33. (c&d) AUC for WT (white bars) and MIF^−/−^ (black bars) mice over the course of GTT and ITT expressed as arbitrary units (AU). Lean n = 9, obese n = 18–33. (e) Plasma insulin levels over time in response to glucose challenge (white circles = WT lean; black circles = MIF−/− lean; white squares = WT obese; black squares = MIF−/− obese). Lean n = 9, obese n = 9. (f) Accumulative weight of WT (white circles) and MIF^−/−^ (black circles) mice. (g) Accumulative food intake of WT (white circles) and MIF^−/−^ (black circles). (h) Body mass composition (white bars = lean fat mass; black bars = fat fat mass). Lean n = 3–4, obese n = 8–9. Data are mean ± SEM, *p<0.05, **p<0.01 and ***p<0.001 w.r.t obese WT.

The severity of HFD-induced IR was less in obese MIF^−/−^ mice, with significantly lower GTT and ITT compared to obese WT mice (Figure 1A–D). Fasting plasma insulin levels increased following the HFD irrespective of genotype, however obese MIF^−/−^ mice secreted significantly less insulin in response to glucose compared to obese WT mice (Figure 1E). Baseline GTT and ITT were not different between WT and MIF^−/−^ mice. Age-matched chow-fed WT and MIF^−/−^ mice GTTs and ITTs were equivalent and significantly lower than obese WT and MIF^−/−^ mice (Figure S1A–D). Despite equivalent body weight at baseline and comparable food intake during the intervention, MIF^−/−^ mice gained significantly less weight than WT mice in response to HFD (Figure 1F–G). Body mass composition confirmed the lower body weight was due to reduced fat mass (Figure 1H), however we could not determine the body regions of fat distribution. Nevertheless, we measured the weight of various organs and observed both liver and epididymal weights were significantly greater in obese WT compared to MIF^−/−^ mice (Table 1). Histological analysis confirmed obese MIF^−/−^ mice display a hyperplasic morphology (Figure S2A). Since weight is a key determinant of insulin sensitivity, we sought to distinguish between direct effects of MIF deficiency on insulin sensitivity from secondary effects of reduced weight. Weight-matched (45–47 g) obese MIF^−/−^ mice had significantly lower GTT and ITT, compared to equivalently obese WT mice, indicating that improved glucose homeostasis in obese MIF^−/−^ mice is independent of body weight (Figure S2B–E). Fasting plasma triacylglycerol (TAG), NEFA, cholesterol, IL-6 IL-10, IL-12p70 and MCP-1 levels increased equivalently in both genotypes following HFD compared to lean counterparts. Plasma leptin and keratinocyte chemoattractant (KC) levels were lower in HFD MIF^−/−^ mice compared WT mice following feeding of a HFD (Table 1).

**Table 1:** Plasma metabolic profile and adipose tissue depot weights.

	WT lean	WT obese	MIF^−/−^lean	MIF^−/−^ obese
Leptin (ρg/ml)	1260.63±189.18	^###^31,695.28±4574.54	2141.74±564.37	^##^18876.88±3570.48*
IL6 (ρg/ml)	34.28±5.36	78.82±16.64	38.90±7.33	53.26±8.26
IL-10 (ρg/ml)	13.78±3.57	28.35±10.15	15.76±3.46	17.05±3.9
IL-12p70 (ρg/ml)	41.03±8.77	287.76±172.05	46.38±12.78	61.27±11.06
MCP-1(ρg/ml)	43.58±3.36	56.88±5.9	50.54±5.18	46.478±4.33
KC(ρg/ml)	81.90±13.07	^###^227.55±24.88	133.87±40.10	145.01±17.12**
NEFA (mmol/l)	22.93±4.71	12.3±2.13	10.72±2.06	12.51±1.83
TAG (mmol/l)	91.28±12.88	137.2±11.46	80.50±3.09	123.36±9.19
Cholesterol(mmol/l)	49.73±2.69	114.74±7.07	48.03±11.41	100.03±8.82
EAT weights	0.32±0.04	1.71±0.06	0.34±0.05	1.48 ±0.14*

Plasma was isolated from overnight fasted WT and MIF^−/−^ mice by cardiac puncture and metabolic markers were analyzed enzymatically. (*p<0.05, **p<0.01 w.r.t. WT obese; #p<0.05, ##p<0.01 and ###p<0.001 w.r.t. w.r.t. respective lean).

### Adipose tissue MIF expression is elevated in obese WT mice while lack of MIF reduced ATM recruitment following HFD

**Figure 2:**
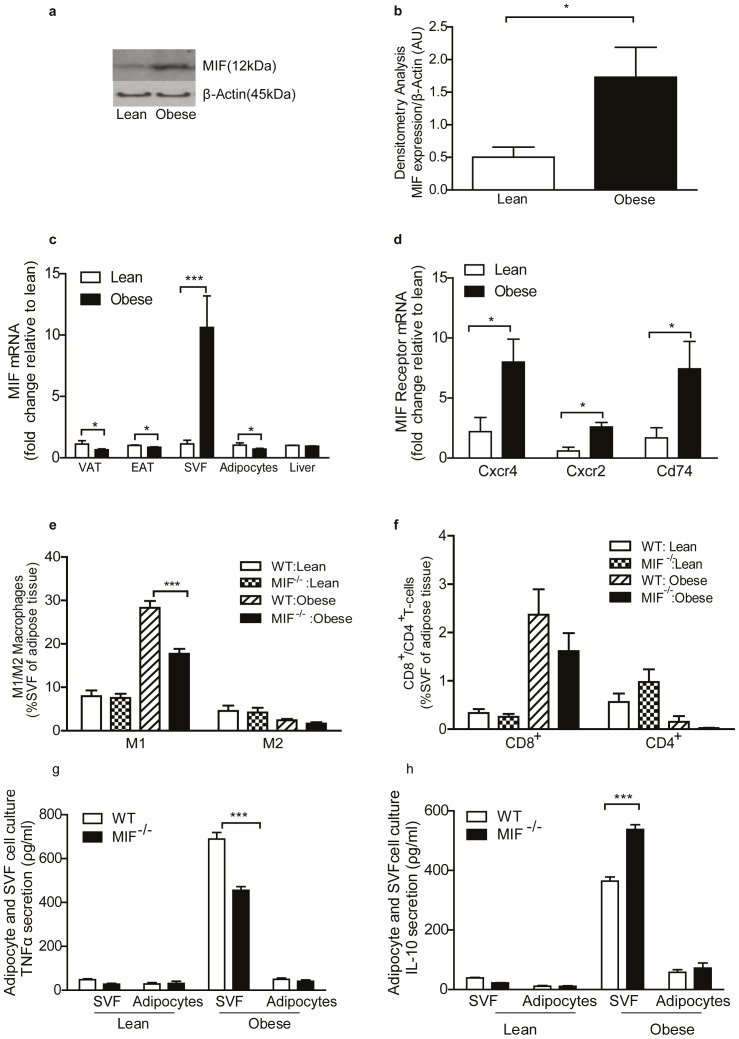
MIF mRNA is elevated in adipose tissue of obese WT mice, while adipose tissue lack of MIF reduced ATM recruitment into adipose tissue following HFD. (a) Immunoblot analysis of MIF and control β-actin in adipose of lean and obese WT mice (n = 4). (b) Densitometry analysis quantifying phosphorylated MIF protein expression relative to β-actin expressed in arbitrary units (AU) in lean (white bars) and obese (black bars) mice. (c) Gene expression analysis determined by RT-PCR of *Mif* in the epididymal adipose tissue (EAT), visceral adipose tissue (VAT), stromal vascular fraction (SVF), adipocyte fraction and liver of lean (white bars) and obese (black bars) WT mice. Lean n = 3–6, obese n = 4–7. (c) Gene expression analysis of known MIF receptors *Cxcr4, Cxcr2, Cd74* in EAT of lean (white bars) and obese (black bars) mice, lean = 4–8, obese n = 3–5. (d) Recruitment of ATM into adipose tissue: cells triple positive (F4/80^+^/CD11B^+^/CD11C^+^) were classified as M1 macrophages. Cells double positive (F4/80^+^/CD11B^+^/CD11C^−^) were classified as M2 macrophages. (e) Recruitment of T-cells into adipose tissue: Cells double positive (CD3^+^/CD4^−^/CD8^+^) were classified as cytotoxic T-cells. Cells double positive (CD3^+^/CD4^+^/CD8^−^) were classified as helper T-Cells. Recruitment of cells is presented as percentage of total SVF cells. Lean WT (white bars) = 9, lean MIF^−/−^ (grey bars) = 9, obese WT (dark grey bars) n = 15–17, obese MIF^−/−^ (black bars) n = 15–17. (f) TNFα and (g) IL-10 cytokine secretion from SVF and adipocyte fraction from lean and obese WT (white bars) and MIF^−/−^ (black bars). Lean = 12, obese n = 12. Data are mean ± SEM, *p<0.05, **p<0.01 and ***p<0.001 w.r.t obese WT.

To confirm the positive relationship between obesity and heightened levels of adipose tissue MIF, we profiled the expression of MIF and its corresponding receptors. Obese EAT expressed significantly higher protein MIF levels compared to lean EAT (Figure 2A–B). Intriguingly, obese EAT and visceral adipose tissue have exhibited reduced *Mif* mRNA expression compared to lean adipose tissue (Figure 2C). In terms of defining the primary cellular source (adipocyte or SVF) of the enhanced adipose tissue MIF, we demonstrated that *Mif* mRNA expression was markedly increased in the adipose SVF but not adipocyte fraction of the obese mice compared to lean mice (Figure 2C). In contrast, *Mif* expression was not altered in liver in response to HFD (Figure 2C). Further gene expression analysis revealed expression of known MIF receptors *Cxcr2, Cxcr4 and Cd74* was significantly up-regulated in the EAT in response to HFD (Figure 2D). Protein levels of CXCR2, CXCR4 and CD74 could not be detected by immunoblot analysis. This may be attributed to reduced levels of stromal vascular cells in whole adipose tissue sections.

**Figure 3:**
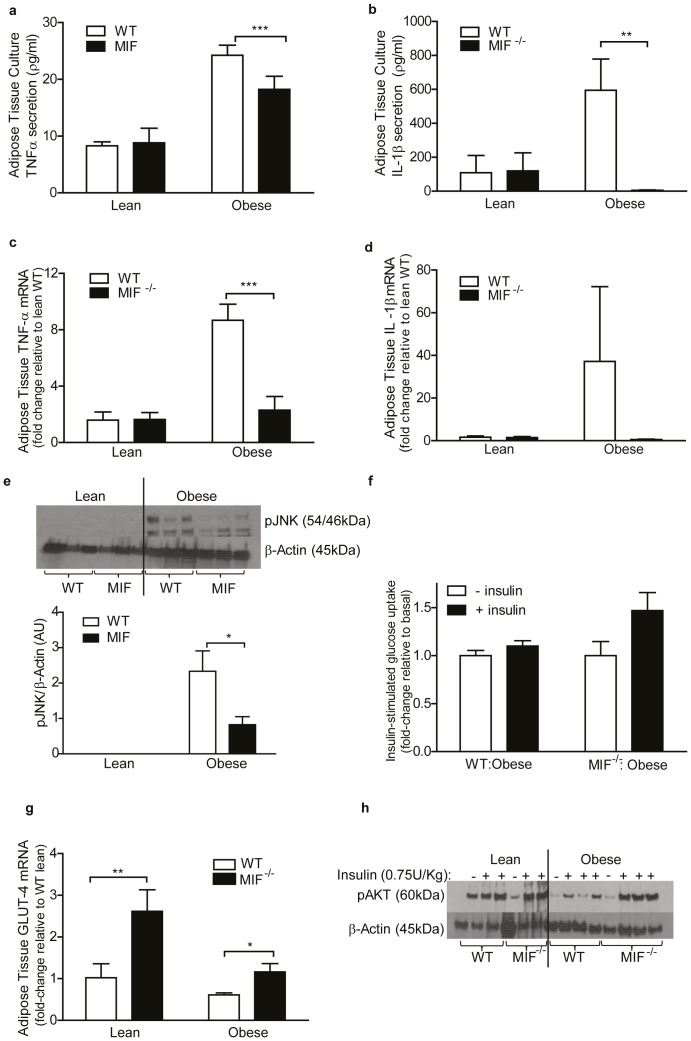
Obese MIF^−/−^ mice exhibit reduced adipose tissue inflammation and improved adipose tissue insulin sensitivity compared to obese WT mice. Levels of pro-inflammatory (a) TNFα and (b) IL-1β secretion into media from adipose tissue explants was measured by ELISA. Lean = 17, obese n = 17. Gene expression analysis of (c) *Tnfα*, (d) *Il-1β* in lean and obese adipose tissue from WT and MIF^−/−^ mice. Lean = 5, obese n = 5. (e) Immunoblot analysis of phosphorylated JNK and control β-actin and corresponding densitometry analysis expressed in arbitrary units (AU) Lean = 3, obese n = 3. (f). *Ex vivo* insulin (100 nM)-stimulated ^3^H-glucose transport into whole adipose tissue (50 mg) harvested from obese WT and MIF^−/−^ mice was evaluated. Fold increase in ^3^H-glucose transport into adipose in response to insulin over basal (non-insulin-stimulated) is presented (white bars =  −insulin, black bars =  + insulin) n = 6. Gene expression analysis of (g) *Glut-4* in adipose tissue from lean and obese WT and MIF^−/−^ mice. Lean = 5, obese n = 5. (h) Immunoblot analysis of phosphorylated AKT levels and control β-actin. Lean = 3, obese n = 3. WT mice represented by white bars, MIF^−/−^ mice represented by black bars in all graphs. Data are mean ± SEM, *p<0.05, **p<0.01 and ***p<0.001 w.r.t obese WT.

Next, we speculated that a reduction in adipose tissue immune cell infiltration would explain the attenuated IR phenotype observed in MIF^−/−^ mice. Both SVF and whole adipose tissue *F4/80* mRNA expression were decreased in MIF^−/−^ mice (Figure 3SA–B). Concomitant, the recruitment of M1 ATM in response to HFD was markedly reduced in obese MIF^−/−^ mice compared to obese WT (Figure 2E). M2 ATM number decreased in response to the HFD, irrespective of genotype (Figure 2E). Correspondingly, M2 ATM marker *Cd206* was equivalently expressed in adipose of WT and MIF^−/−^ mice (Figure S3B). No difference in either CD8^+^ or CD4^+^ T-cell number was evident between genotypes (Figure 2F). The inflammatory signature of SVF displayed a reduction in TNFα (Figure 2G) and IL-1β (Figure S3c) secretion compared to obese WT. Conversely IL-10 (Figure 2H) and MCP-1 secretion (Figure S3D) was increased in obese MIF^−/−^ SVF; while IL-6 secretion was equivalent between genotypes (Figure S3E). No difference in cytokine secretion was evident between genotypes from lean or obese adipocytes.

### MIF deficiency attenuates adipose tissue inflammation and improves insulin sensitivity *ex vivo*

Adipose tissue inflammation was assessed in explants from lean and obese WT and MIF^−/−^ mice TNFα and IL-1β secretion was significantly lower from obese MIF^−/−^ explants compared to obese WT explants (Figure 3A-B), with reduced *Tnfα* and *Il-1β* mRNA expression in obese MIF^−/−^ adipose tissue (Figure 3C–D). Furthermore, phosphorylated JNK, but not NF*κ*Bp65, p38 and ERK, was significantly reduced in obese MIF^−/−^ versus WT adipose tissue (Figure 3E; Figure S4A–C). Subsequently, we investigated if reduced adipose tissue inflammation translated to improved adipose tissue insulin sensitivity in MIF^−/−^ mice. Insulin-stimulated ^3^H-glucose uptake into adipose explants was reduced in WT obese adipose compared to MIF^−/−^ obese adipose (Figure 3F). Coincident with HFD, *Glut-4* mRNA expression decreased in both genotypes; however *Glut-4* mRNA expression remained higher in obese MIF^−/−^ mice compared to obese WT (Figure 3G). A comparable reduction in adipose *Irs-1* mRNA expression was observed in obese WT and MIF^−/−^ mice following HFD (Figure S4D). To investigate the effect of MIF on insulin signaling lean and obese WT and MIF^−/−^ animals were injected with or without insulin. Obese MIF^−/−^ adipose tissue displayed markedly increased levels of phosphorylated AKT compared to corresponding obese WT adipose tissue (Figure 3H).

### MIF^−/−^ BMM have improved adipocyte-macrophage crosstalk, while ISO-1 ameliorates the adverse effects of MIF on adipocyte insulin sensitivity

**Figure 4:**
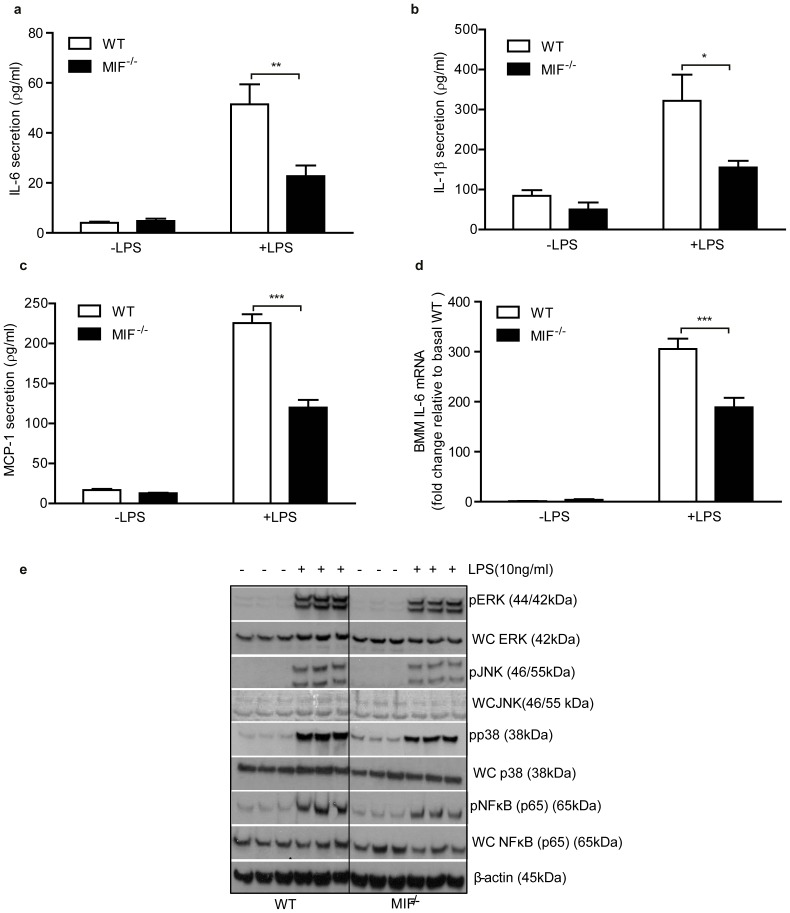
MIF^−/−^ bone marrow macrophages have reduced inflammatory signature compared to WT mice. Levels of pro-inflammatory (a) IL-6, (b) IL-1β (c) MCP-1 secretion into media from lean WT and MIF^−/−^ BMM stimulated ±LPS. (d) Gene expression analysis of *Il-6* in WT and MIF^−/−^ BMM±LPS, n = 5/group. (e) Immunoblot analysis of phosphorylated and whole cell ERK, JNK, p38, NFkB and β-actin stimulated ±LPS, n = 3/group. WT mice represented by white bars, MIF^−/−^ mice represented by black bars in all graphs. Data are mean ± SEM, *p<0.05, **p<0.01 and ***p<0.001 w.r.t WT+LPS.

**Figure 5:**
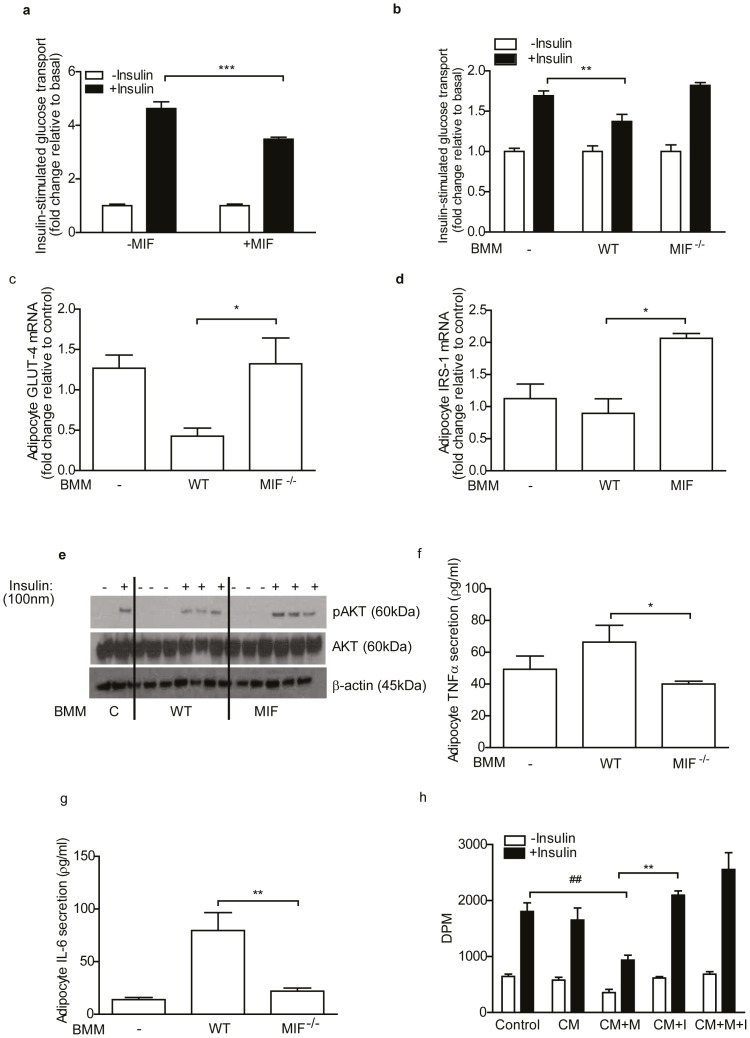
MIF^−/−^ macrophages have altered adipocyte-macrophage crosstalk compared to WT macrophages, while ISO-1 blocks MIFs insulin desensitizing capacity in adipocytes. (a) Chronic treatment of 3T3-L1 adipocytes with rMIF (100 ng) and its effect on insulin (100 nM)-stimulated ^3^H-glucose uptake was evaluated. Data are mean ± SEM, *p<0.05, **p<0.01 and ***p<0.001 w.r.t untreated +Insulin. (b) The effect of WT and MIF^−/−^ BMM on insulin (100 nM)-stimulated ^3^H-glucose transport into 3T3-L1-adipocytes was evaluated. Fold increase in ^3^H-glucose transport into adipocytes in response to insulin over basal (non-insulin-stimulated) is presented (white bars =  -insulin, black bars =  + insulin). Data are mean ± SEM, *p<0.05, **p<0.01 and ***p<0.001 w.r.t adipocytes co-cultured with WT BMM+insulin. The effect of BMM co-culture on adipocyte on (c) *Glut-4* and (d) *Irs-1* mRNA expression was determined by real-time PCR, n = 4/group. (e) Immunoblot analysis of phosphorylated AKT, whole-cell AKT and β-actin in co-cultured adipocytes stimulated with insulin (100 nM). Levels of (f) TNFα (g) IL-6, were measured in media from co-cultured cells. N = 4/group, data are mean ± SEM, *p<0.05, **p<0.01 and ***p<0.001 w.r.t adipocytes co-cultured with WT BMM. (h) The effect of unstimulated J774.2 CM, MIF (100 ng/ml)-stimulated (CM+M), ISO-1 (50 µMl) treated (CM+I) and cells pretreated with ISO-1 (50 µM) 1 hour prior MIF-stimulation (CM+M+I) on insulin (100 nM)-stimulated ^3^H-glucose transport into 3T3-L1adipocytes was evaluated and expressed in DPM. N = 4/group, data are mean ± SEM, *p<0.05, **p<0.01 and ***p<0.001 w.r.t adipocytes treated with (CM+M), #p<0.05, ##p<0.01 and ###p<0.001 w.r.t control.

Given the improved immunophenotype observed in obese MIF^−/−^ SVF we hypothesized that MIF^−/−^ macrophages would have an attenuated pro-inflammatory phenotype and improve cross-talk with adipocytes *in vitro.* We determined the effect of MIF^−/−^ BMM on adipocyte biology as a surrogate for ATM; as the number of ATM were limited for mechanistic studies. MIF^−/−^ BMM secreted significantly less IL-6, IL-1β and MCP-1 and exhibited reduced *Il-6* and *iNos* mRNA expression compared to WT (Figure 4A–D; Figure S3E). While anti-inflammatory IL-10 demonstrates a marked increase in MIF^−/−^ BMM compared to WT (Figure S3F). Furthermore, activation of MAPKs; p38, JNK, ERK, and NFκB pathways was significantly impaired in MIF^−/−^ BMM compared to WT (Figure 4E). Exogenous rMIF directly impaired insulin-stimulated glucose transport in 3T3-L1-adipocytes (Figure 5A) which corroborates previous work [33]. Furthermore we demonstrated that 3T3-L1-adipocytes, co-cultured with WT BMM, but not with MIF^−/−^ BMM, had reduced adipocyte insulin-stimulated glucose uptake, with decreased *Glut-4* and *Irs-1* mRNA expression (Figure 5B–D). Co-culture of adipocytes with WT BMM significantly reduced insulin-stimulated phosphorylation of AKT, an effect which was not observed when co-cultured with MIF^−/−^ BMM (Figure 5E). Also, adipocytes co-cultured with MIF^−/−^ BMM secreted less TNFα and IL-6 compared to adipocytes co-cultured with WT BMM (Figure 5F-G). Lastly we examined if ISO-1, a MIF inhibitor which targets MIFs D-dopachrome tautomerase enzymatic activity would inhibit MIF inflammatory effects in macrophages and improve macrophage-adipocyte crosstalk. Pre-treatment with ISO-1 blocked MIF-induced TNFα cytokine secretion from J774.2 macrophages (Figure S5A). Subsequently, 3T3-L1 adipocytes were incubated with MIF treated CM in the presence or absence of ISO-1. CM from unstimulated macrophages had no effect on insulin-stimulated glucose uptake into adipocytes while pretreatment with ISO-1 reversed the insulin desensitizing effects of MIF in 3T3-L1 adipocytes (Figure 5H).

### Short-term ISO-1 treatment does not impede HFD-induced IR or alter immune cell recruitment

We next assessed if short-term treatment with ISO-1 would mirror our results observed in MIF^−/−^ mice. As obesity is a low grade chronic inflammatory state we first speculated a low dose of 10 mg/kg over 14 consecutive days would have therapeutic benefits [34], [35]. ISO-1 treated animals exhibited similar glucose tolerance to vehicle control treated animals (Figure S5B). As such a higher dose was proposed. A subset of WT mice were treated with 35 mg/kg ISO-1 daily for 3 days. Insulin sensitivity was comparable between ISO-1 treated and saline treated groups (Figure S5C). ISO-1 treated and saline treated animal's recruited equivalent numbers of M1 and M2 ATM following a HFD (Figure S5D). Overnight cultured SVF harvested from ISO-1 treated and vehicle control groups secreted equivalent levels of TNFα, and IL-1β (Figure S5E–F).

### MIF deficiency alleviates hepatic steatosis and improves hepatic insulin sensitivity in response to HFD

**Figure 6:**
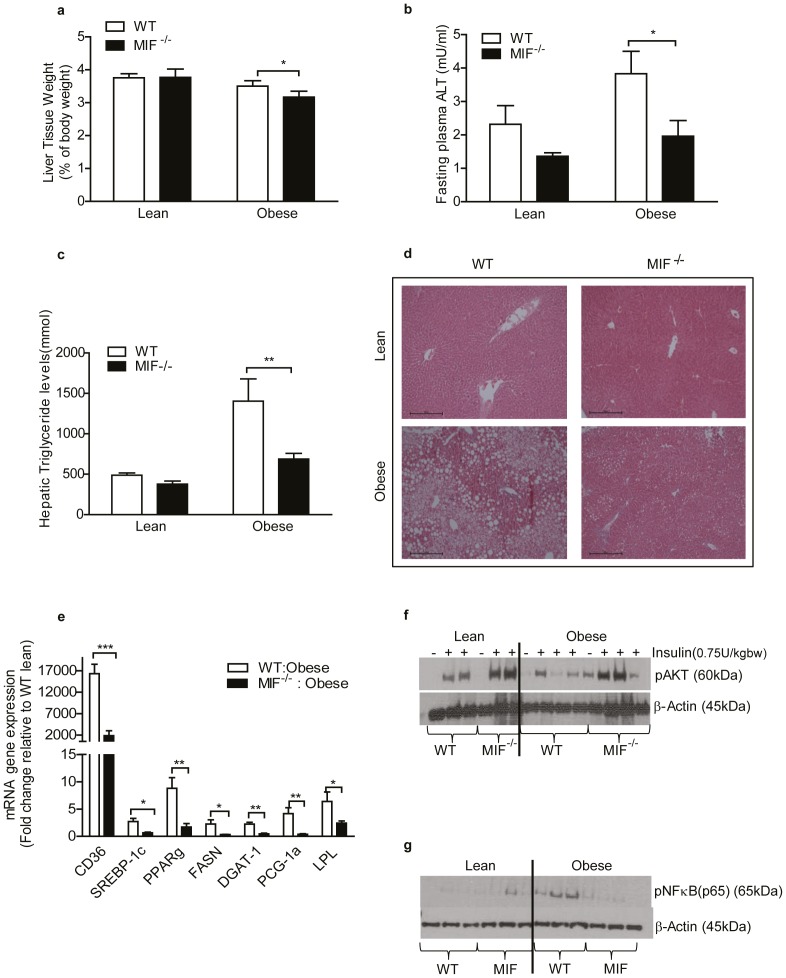
Lack of MIF improves HFD-induced hepatic steatosis and hepatic insulin sensitivity. Liver tissue was harvested from lean and obese WT and MIF^−/−^ mice. (a) Weight of liver tissue expressed as a percentage of total body weight. Lean = 4, obese = 4. (b) Fasting plasma ALT levels in lean and obese mice. (c) Hepatic triacylglyceride levels, lean = 4, obese = 4. (d) Hematoxylin & eosin staining to visualize hepatic lipid accumulation (representative of n = 3 images per group). (e) Gene expression analysis of markers of lipogenesis and lipid storage as determined by RT-PCR. Lean = 4, obese = 4. (f) Immunoblot analysis of phosphorylated AKT and β-actin from livers of WT and MIF^−/−^ mice ± insulin. Lean = 3, obese = 3. (g) Unstimulated tissue phosphorylated-NFkB were determined by immunoblot analysis. Lean = 3, obese = 3. WT mice represented by white bars, MIF^−/−^ mice represented by black bars in all graphs. Data are mean ± SEM, *p<0.05, **p<0.01 and ***p<0.001 w.r.t obese WT.

Adipose tissue expansion exacerbates lipolysis and elevates NEFA influx into liver. MIF^−/−^ mice had reduced liver weight compared to WT mice following HFD indicating partial protection from HFD-induced hepatomegaly (Figure 6A) Furthermore, fasting plasma alanine aminotransaminase (ALT) levels were considerably lower in obese MIF^−/−^ mice compared to obese WT, indicative of reduced liver tissue injury (Figure 6B). Hepatic TAG content was significantly lower in obese MIF^−/−^ mice compared to obese WT mice (Figure 6C). Hematoxylin and eosin (H&E) studies confirmed that obese MIF^−/−^ mice have reduced lipid accumulation compared to obese WT after HFD (Figure 6D). Also lipogenic genes *Cd36, Dgat-1, Fasn, Srebp-1c, Pgc-1α, Lpl and Pparγ* mRNA expression was significantly lower in obese MIF^−/−^ mice compared to obese WT (Figure 6E). Lastly, we investigated the effect of MIF on hepatic insulin signaling and inflammation. Insulin-stimulated hepatic phosphorylated AKT expression was significantly greater in both lean and obese MIF^−/−^ mice compared to WT controls (Figure 6F). Furthermore phosphorylated NFκBp65 levels were markedly lower in obese MIF^−/−^ mice compared to obese WT mice (Figure 6G)

## Discussion

Expanding adipose tissue mass in response to HFD is accompanied by a progressive infiltration of macrophages and T-cells which contribute significantly to inflammation and IR [4]. The mediators which initiate ATM recruitment remain to be fully deciphered. Here we have shown that HFD augments local adipose tissue MIF expression, which is primarily attributed to the adipose SVF. We demonstrate that MIF is pivotal for HFD-induced ATM recruitment and adipose tissue inflammation. Impeding MIF with ISO-1 attenuates MIFs pro-inflammatory, insulin desensitizing effects in a macrophage-adipocyte conditioned media system. However the beneficial effects of ISO-1 were not evident *in vivo.* Furthermore MIF deficiency partially protects from HFD-induced obesity, IR and hepatic steatosis.

MIF affects glucose metabolism at several levels, including pancreatic insulin secretion [36], TNFα mediated adipocyte IR and glucose uptake and glycolysis in skeletal muscle [37]. Recent reports present conflicting data regarding its role in glucose homeostasis *in vivo*. Deletion of MIF in LDLR^−/−^ mice impeded the development of atherosclerosis, glucose intolerance and IR without altering adiposity [32]. We extend this knowledge by demonstrating that obese MIF^−/−^ mice are partially protected from HFD-induced IR, are less hyperinsulinemic in response to a glucose load and exhibit improved glucose homeostasis. Increased levels of MIF can enhance insulin secretion in a glucose dependent manner [36] and this may in turn explain the reduced hyperinsulinemia observed in obese MIF^−/−^ mice. Consistent with Verschuren *et al.,* findings MIF^−/−^ mice display adipocyte hyperplasia. However unlike Verschuren *et al* we illustrate MIF^−/−^ mice exhibit reduced weight gain attributable to lower fat mass, without altered feeding behavior in response to HFD. Plasma insulin levels in chow fed animals are equivalent, suggesting that absence of MIF does not impair insulin production/secretion but reduction may be reflective of reduced weight gain and improved insulin sensitivity in HFD-fed MIF^−/−^ mice. Improved glucose tolerance was still evident in obese MIF^−/−^ mice compared to weight-matched obese WT, thus we speculate that MIF has additional beneficial mechanisms beyond reduced weight-gain. Conversely, Serre-Beiner *et al.* showed that absence of MIF led to impaired glucose tolerance, hyperinsulinemia and increased body fat mass in mature mice compared to WT mice in response to chow diet [38], however interactions with HFD were not examined. These contradictory findings underscore the complexity of MIF inflammatory signals within glucose and energy metabolism depending on diet and age. Further, increases in plasma leptin levels observed in the obese state are usually directly proportional to increases in plasma insulin levels and expanding adipose tissue mass [39]. It is therefore likely that the reduced adipose tissue mass in MIF^−/−^ mice accounts for differences in plasma leptin levels.

ATM infiltration and inflammation are crucial to the pathogenesis of HFD-induced IR. Ablation of pro-inflammatory CD11c^+^(M1) macrophages ameliorates IR coincident with diminished systemic inflammation in obese mice [40]. Furthermore mice lacking PPARγ in myeloid cells exhibit reduced numbers of M2 macrophages and are susceptible to HFD-induced IR [41]. We postulated that given the potent chemotactic properties of MIF [31], reduced ATM accumulation would explain the partial protection from HFD-induced IR observed in MIF^−/−^ mice. In support of our hypothesis, M1 ATM number was lower in obese MIF^−/−^ adipose tissue compared to WT coincident with reduced adipose F4/80, while M2 ATM number was equivalent. Our study demonstrated reduced circulating levels of keratinocyte-derived chemokine (KC), a mouse ortholog for human IL-8, in MIF^−/−^ mice after HFD compared to WT mice. KC signals via a known MIF receptor CXCR2 which is involved in chemotaxis. It is plausible that the combination of reduced KC and lack of MIF contributed to reduced inflammatory cell recruitment into MIF^−/−^ adipose tissue and preservation of insulin sensitivity. Furthermore, a previous study by Lin et al., demonstrated that administration of exogenous MIF increased circulating KC in mice indicative of a direct capacity of MIF to regulate KC levels [42].

Obese MIF^−/−^ SVF secreted less TNFα and IL-1β compared to WT SVF. IL-10 secretion was increased from MIF^−/−^ SVF compared to WT SVF. In contrast lack of MIF did not alter adipose T-cell CD8^+^ or CD4^+^ populations as illustrated in Figure 2F. Recent studies have implicated the immunogenic phenotype of ATM is of critical importance in dictating the severity of adipose tissue inflammation and IR [43]. We speculated that the improved inflammatory profile was macrophage derived, however given that we are limited to interpreting the specific cellular source of cytokines while working within the mixed cell pool of the SVF, we investigated the response of a more purified macrophage population from WT and MIF^−/−^ mice to an LPS stimulus by utilizing BMM. Furthermore, MIF^−/−^ macrophages have previously displayed an attenuated inflammatory phenotype in response to the TLR4 ligand LPS *in vitro* [44], [45]. Correspondingly MIF^−/−^ BMM demonstrated improved immunogenic phenotype with reduced expression of M1 marker iNOS and heightened IL-10 secretion. Furthermore co-culture of MIF^−/−^ BMM with adipocytes resulted in reduced inflammation and preservation of adipocyte insulin sensitivity compared to co-culture with WT BMM. This data highlights the significance of MIF in the pathophysiology of HFD-induced IR not only as controller of M1 ATM macrophage recruitment but also as a modulator of macrophage activation status and subsequent insulin de-sensitizing capacity. Recently, Saksida and co-workers showed inhibition of MIF *in vitro* utilizing MIF inhibitor ISO-1 reduced palmitic acid-induced pancreatic beta cell dysfunction [46]. We also have highlighted the potential therapeutic potential of MIF inhibitors by demonstrating that ISO-1 successfully blocked MIF-induced macrophage inflammation and reversed the detrimental effects of MIF-stimulated macrophages on adipocyte function *in vitro*. On the contrary, short term treatment with ISO-1 failed to attenuate HFD-induced IR and or impede immune cell infiltration *in vivo*. It is probable that the timing and dosing of the ISO-1 inhibitor accounted for lack of efficacy in the obese phenotype. An alternative MIF antibody with greater specificity may provide greater efficacy.

Several models of inflammation have indicated MIF is a central controller of systemic inflammation [21], [47], [48]. This study extends our current understanding of MIF as an inducer of local inflammation within the context of HFD-induced adipocyte dysfunction. We hypothesized that reduction of M1 ATM number and diminished immunogenic phenotype of adipose tissue SVF in obese MIF^−/−^ mice may attenuate whole adipose tissue inflammation and further dissipate adipocyte dysfunction. Obese MIF^−/−^ whole adipose tissue cultured *ex vivo* exhibited a marked reduction in TNFα and IL-1β secretion accompanied by reduced *Tnfα* and *Il-1β* mRNA expression compared with obese WT. Moreover JNK activation was impaired in obese MIF^−/−^ adipose tissue, while no difference in pNFκBp65 or pp38 activity was observed. Attenuated TNFα and IL-1β secretion may account for the observed increase in JNK activity. Interestingly, deletion of JNK-1 in obese mice reduced adiposity and alleviated IR [49], we could therefore speculate that diminished JNK activation in obese MIF^−/−^ adipose tissue may contribute to reduced adiposity. Concomitant with attenuated local adipose tissue inflammation and corroborating our hypothesis we observed improved insulin sensitivity in obese MIF^−/−^ adipose tissue, whereby in insulin-stimulated ^3^H-glucose uptake into adipose explants was increased in obese MIF^−/−^ compared to WT mice.

The contribution of MIF to HFD-induced hepatic steatosis has been relatively unexplored. We demonstrate that obese MIF^−/−^ mice have reduced hepatomegaly, lower systemic ALT levels and are partially protected from HFD-induced hepatic steatosis; coincident with reduced *Pparγ* and *Srebp-1c* mRNA expression compared to WT. *Pparγ* regulates expression of the fatty acid translocase protein *Cd36*, expression of which is also reduced in obese MIF ^−/−^ mice compared to WT. Previous studies have reported that lean mice lacking MIF have improved hepatic insulin sensitivity [38]. In addition, we demonstrated that obese MIF^−/−^ liver tissue has heightened insulin sensitivity compared to obese WT. Overflow of NEFA from obese, insulin resistant adipose tissue to liver contributes to hepatic steatosis and resultant IR, an effect which is markedly improved upon disruption of ATM recruitment [50], [51] [12]. Whether MIF directly induces hepatic lipid accumulation or indirectly via ATM recruitment is yet to be established.

In conclusion, this study illustrates a direct role for MIF signaling in HFD-induced ATM recruitment, adipose dysfunction and glucose homeostasis. However, there were limitations to our study. These studies were performed in whole-body MIF deficient mice, whether lack of MIF within the immune or non-immune system is primarily governing these protective effects remains to be addressed. Furthermore the significant reduction in body weight warrants further investigation to decipher whether this is an intrinsic characteristic of this mouse model or if MIF regulates energy homeostasis and/or gut assimilation. Nevertheless, given the body of evidence presented, we speculate that small molecule MIF inhibitors may have therapeutic potential to ameliorate obesity-induced IR, halting the progression to T2D and non-alcoholic fatty liver disease.

## Supporting Information

Figure S1 **Glucose tolerance (GTT) and insulin tolerance tests (ITT) in WT and MIF^−/−^ mice after high fat and after chow diet. (**A) GTT (1.5 g/kg glucose) in 4–6 hour fasted age-matched chow-fed lean and high-fat fed obese Wild-Type (WT) and MIF^−/−^ animals (black circles = WT lean; open circles =  MIF^−/−^ lean; black squares = WT obese; open squares = MIF^−/−^ obese; *p<0.05, **p<0.01 w.r.t. MIF^−/−^ obese; n = 9). (C) ITT (0.75 U/kg insulin) in 6 h fasted lean and obese WT and MIF^−/−^ animals (black circles = WT lean; open circles = MIF^−/−^ lean; black squares = WT obese; open squares = MIF^−/−^ obese, *p<0.05 w.r.t. MIF^−/−^ obese, n = 7–9). (B&D) Area under the curve (AUC) for lean and obese animals over course of GTT and ITT was calculated and expressed as arbitrary units (AU), (*p<0.5,^ ***^p<0.001 w.r.t. WT, ^##^p<0.01, ^###^p<0.001 w.r.t. corresponding lean, n = 7–9).
(TIF)

Figure S2 **MIF deficiency improves glucose homeostasis in response to HFD in weight-matched animals.** (A) Histological analysis of paraffin embedded adipose tissue (image representation of n = 6/group). (B) GTT (1.5 g/kg glucose) in 6 hour fasted lean and obese WT and MIF^−/−^ animals (n = 9/group; white circles = WT lean; black circles =  MIF^−/−^ lean; white squares = WT obese; black squares = MIF^−/−^ obese; ***p<0.01 w.r.t. MIF^−/−^ obese; n = 9). (B) Area under the curve (AUC) over course of GTT was calculated and expressed as arbitrary units (AU). (C) ITT (0.75 U/kg insulin) in 6 h fasted lean and obese WT and MIF^−/−^ animals (n = 9/lean group, n = 18–33/obese group; white circles = WT lean; black circles = MIF^−/−^ lean; white squares = WT obese; black squares = MIF^−/−^ obese, **p<0.01 w.r.t. MIF^−/−^ obese). (D) Area under the curve (AUC) over course of ITT was calculated and expressed as arbitrary units (AU), (n = 9/lean group, n = 18–33/obese group; *p<0.5, ***p<0.01 w.r.t. obese WT, ^###^p<0.001 w.r.t. corresponding lean).
(TIF)

Figure S3 **Stromal vascular fraction (SVF) inflammatory and adipocyte inflammatory signature is altered in MIF^−/−^ mice compared to WT mice in response to HFD.** (A) *F4/80* mRNA expression in SVC from obese mice only (n = 4/group; ***p<0.001 w.r.t. WT obese). (B) Adipose tissue *F4/80 and Cd206* mRNA expression (n4 = /group, ***p<0.001 w.r.t. WT obese). (C) IL-1β, (D) MCP-1 and (E) IL-6 and cytokine secretion from SVF cells and adipocytes from lean and obese mice cultured in serum rich media for 24 hours (seeded 1million cells/1 ml) (n = 12/group; ***p<0.001 w.r.t. WT obese).
(TIF)

Figure S4 **Adipose tissue inflammation.** (A) Immunoblot analysis of phosphorylated NF*κ*B (p65), (B) p38, (C) ERK and control β-actin in adipose tissue of WT and MIF^−/−^ mice. Densitometry analysis illustrates expression relative to β-actin expressed in arbitrary units (AU) (n = 3/group). (C) *Irs-1* mRNA in adipose tissue of lean and obese animals (n = 5/group). (D) BMM ± LPS (10 ng/ml) *iNos* gene expression (n = 6/group,*p<0.05 w.r.t. WT). (E) BMM ± LPS (10 ng/ml) IL-10 secretion into media measured by ELISA n = 3/group).
(TIF)

Figure S5 **ISO-1 treatment inhibits MIF-induced TNFα cytokine secretion from J774.2 macrophages but cannot restore insulin sensitivity in vivo.** (A) J774.2 macrophages were pre-treated with ISO-1 (50 µM/ml) for 1 hour prior to rMIF (100 ng/ml) stimulation for 3 hours. Media was harvested for TNFα cytokine secretion (n = 3, *p≤0.05, w.r.t MIF stimulated cells,^ #^ p≤0.05, w.r.t control cells. (B) GTT (1.5 g/kg glucose) in 4–6 hour fasted mice treated with or without ISO-1 (n = 10/group). (C) ITT (0.75 U/kg insulin) in 6 hour fasted mice treated with or without ISO-1 (n = 5/group). SVC harvested from mice treated with or without ISO-1 were cultured overnight. (A) TNFα and (E) IL-1β cytokine secretion into media was measured by ELISA (n = 4/group).
(TIF)

File S1 **Supporting materials and methods.**
(DOC)
