# Supplementary figures and images for: Macrophage Migration Inhibitory Factor Deficiency Ameliorates High-Fat Diet Induced Insulin Resistance in Mice with Reduced Adipose Inflammation and Hepatic Steatosis
Source: PLoS One. 2014 Nov 20;9(11):e113369. doi: 10.1371/journal.pone.0113369 (PMC4239060; doi:10.1371/journal.pone.0113369)

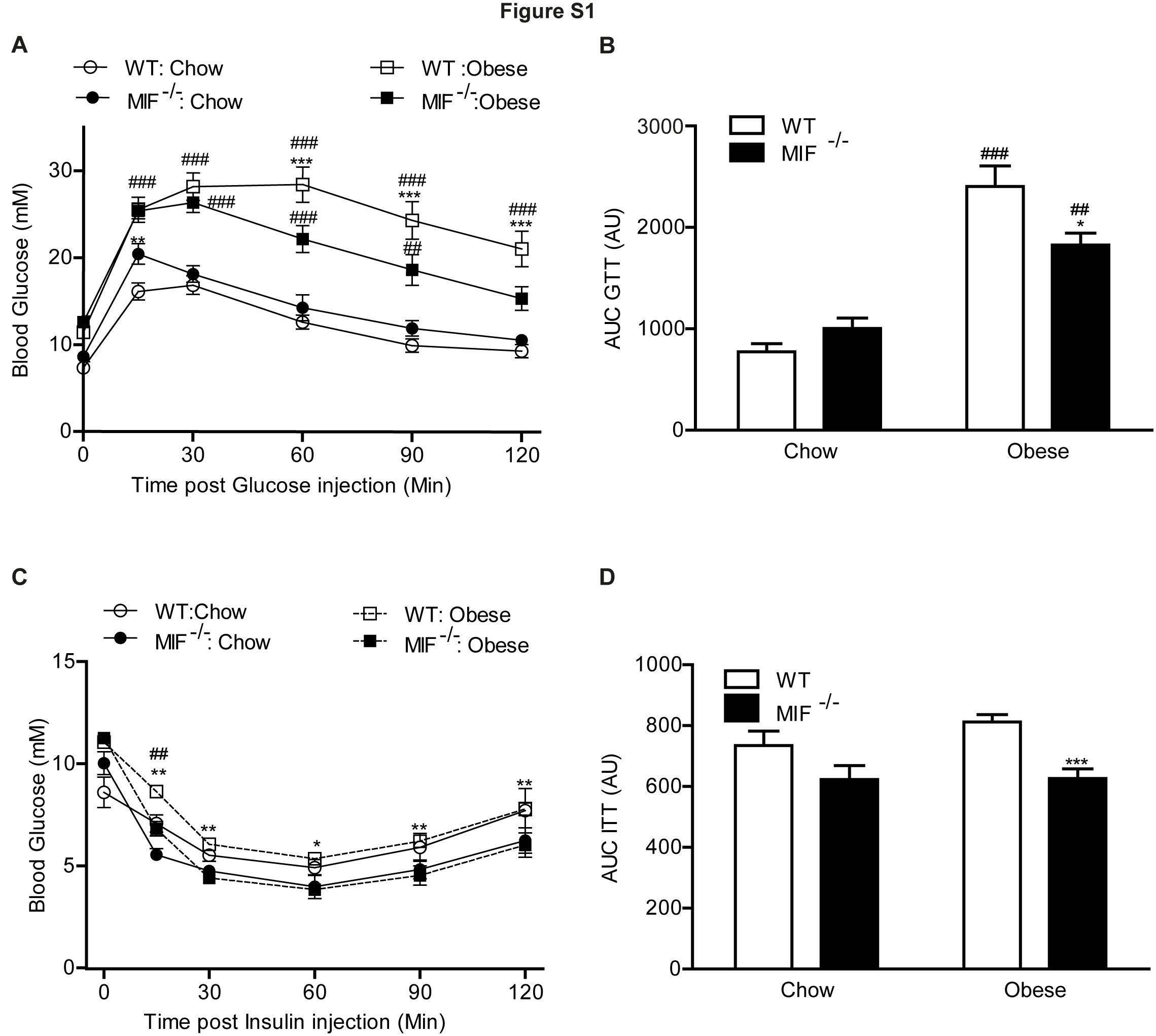

Supplement: Figure S1 — Glucose tolerance (GTT) and insulin tolerance tests (ITT) in WT and MIF−/− mice after high fat and after chow diet. (A) GTT (1.5 g/kg glucose) in 4–6 hour fasted age-matched chow-fed lean and high-fat fed obese Wild-Type (WT) and MIF−/− animals (black circles = WT lean; open circles = MIF−/− lean; black squares = WT obese; open squares = MIF−/− obese; *p<0.05, **p<0.01 w.r.t. MIF−/− obese; n = 9). (C) ITT (0.75 U/kg insulin) in 6 h fasted lean and obese WT and MIF−/− animals (black circles = WT lean; open circles = MIF−/− lean; black squares = WT obese; open squares = MIF−/− obese, *p<0.05 w.r.t. MIF−/− obese, n = 7–9). (B&D) Area under the curve (AUC) for lean and obese animals over course of GTT and ITT was calculated and expressed as arbitrary units (AU), (*p<0.5, ***p<0.001 w.r.t. WT, ##p<0.01, ###p<0.001 w.r.t. corresponding lean, n = 7–9). (TIF) [file pone.0113369.s001.tif]

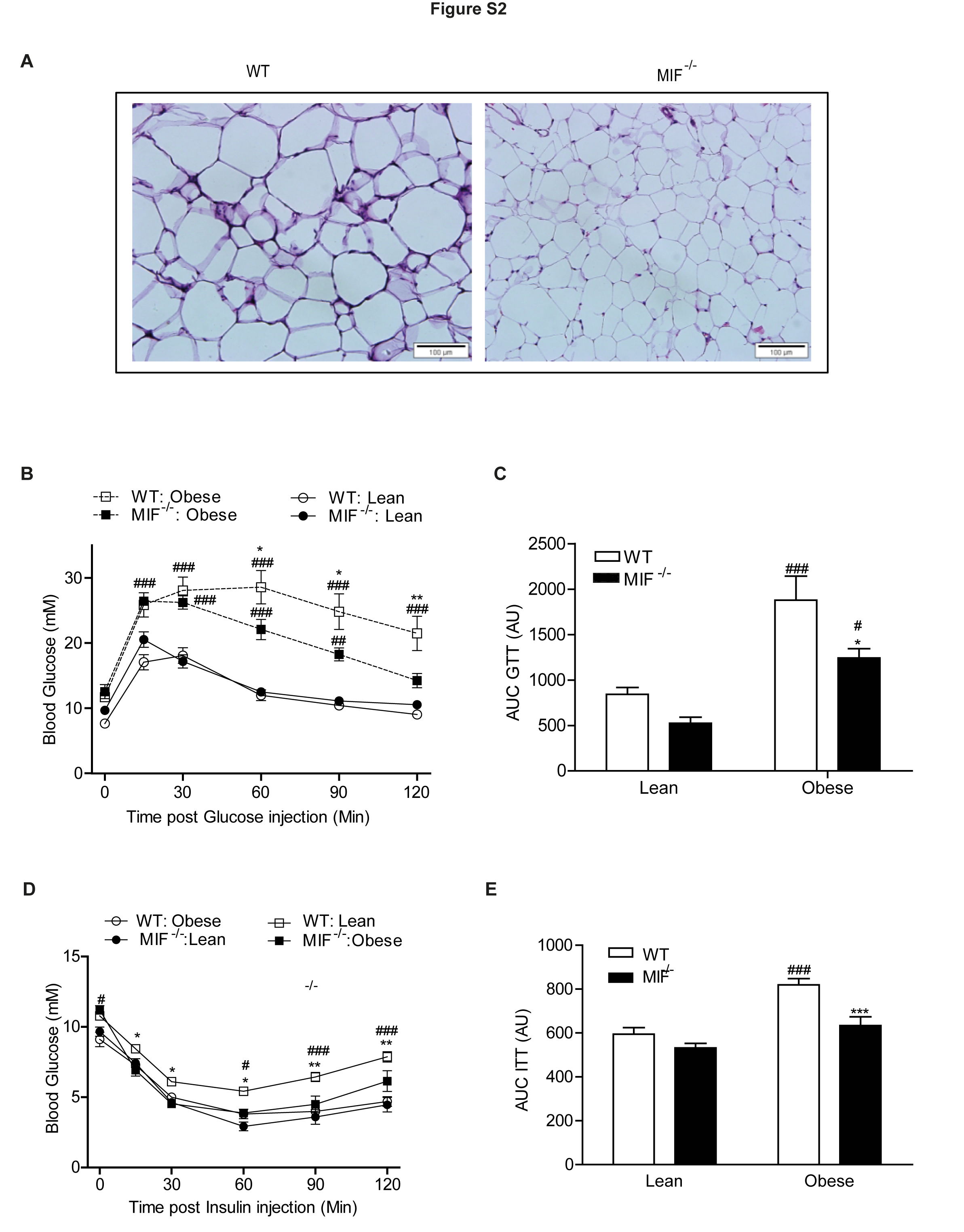

Supplement: Figure S2 — MIF deficiency improves glucose homeostasis in response to HFD in weight-matched animals. (A) Histological analysis of paraffin embedded adipose tissue (image representation of n = 6/group). (B) GTT (1.5 g/kg glucose) in 6 hour fasted lean and obese WT and MIF−/− animals (n = 9/group; white circles = WT lean; black circles = MIF−/− lean; white squares = WT obese; black squares = MIF−/− obese; ***p<0.01 w.r.t. MIF−/− obese; n = 9). (B) Area under the curve (AUC) over course of GTT was calculated and expressed as arbitrary units (AU). (C) ITT (0.75 U/kg insulin) in 6 h fasted lean and obese WT and MIF−/− animals (n = 9/lean group, n = 18–33/obese group; white circles = WT lean; black circles = MIF−/− lean; white squares = WT obese; black squares = MIF−/− obese, **p<0.01 w.r.t. MIF−/− obese). (D) Area under the curve (AUC) over course of ITT was calculated and expressed as arbitrary units (AU), (n = 9/lean group, n = 18–33/obese group; *p<0.5, ***p<0.01 w.r.t. obese WT, ###p<0.001 w.r.t. corresponding lean). (TIF) [file pone.0113369.s002.tif]

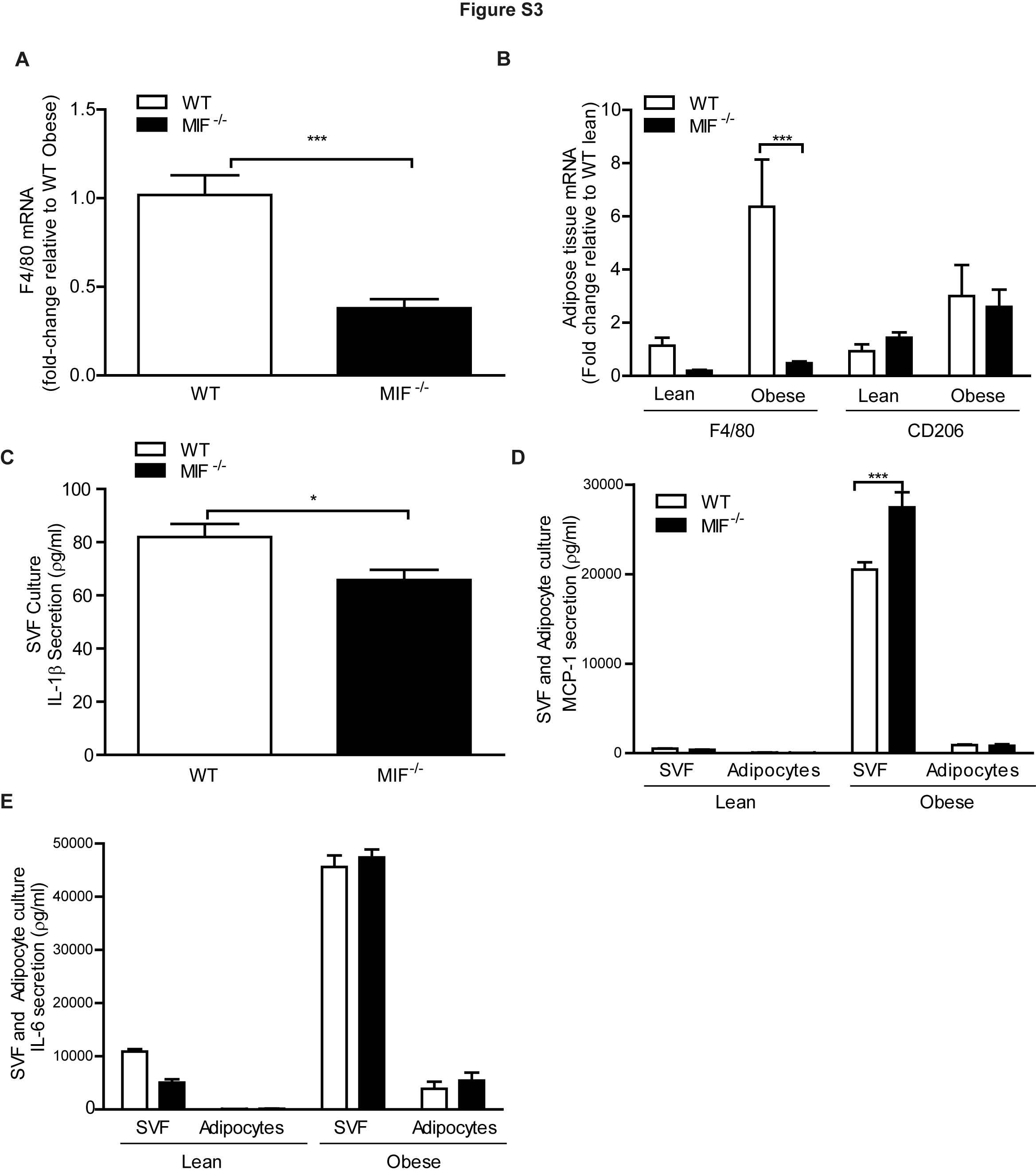

Supplement: Figure S3 — Stromal vascular fraction (SVF) inflammatory and adipocyte inflammatory signature is altered in MIF−/− mice compared to WT mice in response to HFD. (A) F4/80 mRNA expression in SVC from obese mice only (n = 4/group; ***p<0.001 w.r.t. WT obese). (B) Adipose tissue F4/80 and Cd206 mRNA expression (n4 = /group, ***p<0.001 w.r.t. WT obese). (C) IL-1β, (D) MCP-1 and (E) IL-6 and cytokine secretion from SVF cells and adipocytes from lean and obese mice cultured in serum rich media for 24 hours (seeded 1million cells/1 ml) (n = 12/group; ***p<0.001 w.r.t. WT obese). (TIF) [file pone.0113369.s003.tif]

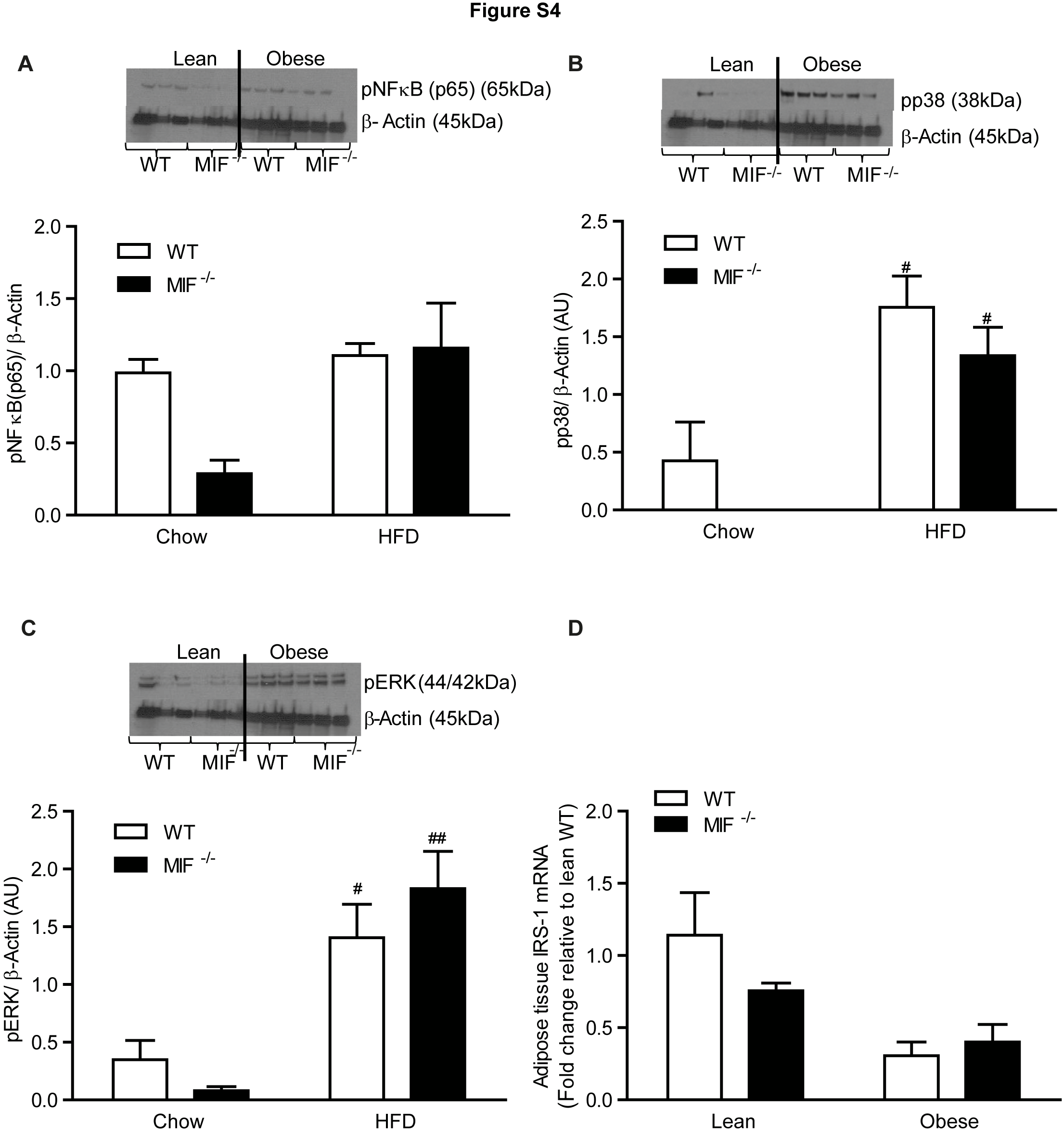

Supplement: Figure S4 — Adipose tissue inflammation. (A) Immunoblot analysis of phosphorylated NFκB (p65), (B) p38, (C) ERK and control β-actin in adipose tissue of WT and MIF−/− mice. Densitometry analysis illustrates expression relative to β-actin expressed in arbitrary units (AU) (n = 3/group). (C) Irs-1 mRNA in adipose tissue of lean and obese animals (n = 5/group). (D) BMM ± LPS (10 ng/ml) iNos gene expression (n = 6/group,*p<0.05 w.r.t. WT). (E) BMM ± LPS (10 ng/ml) IL-10 secretion into media measured by ELISA n = 3/group). (TIF) [file pone.0113369.s004.tif]

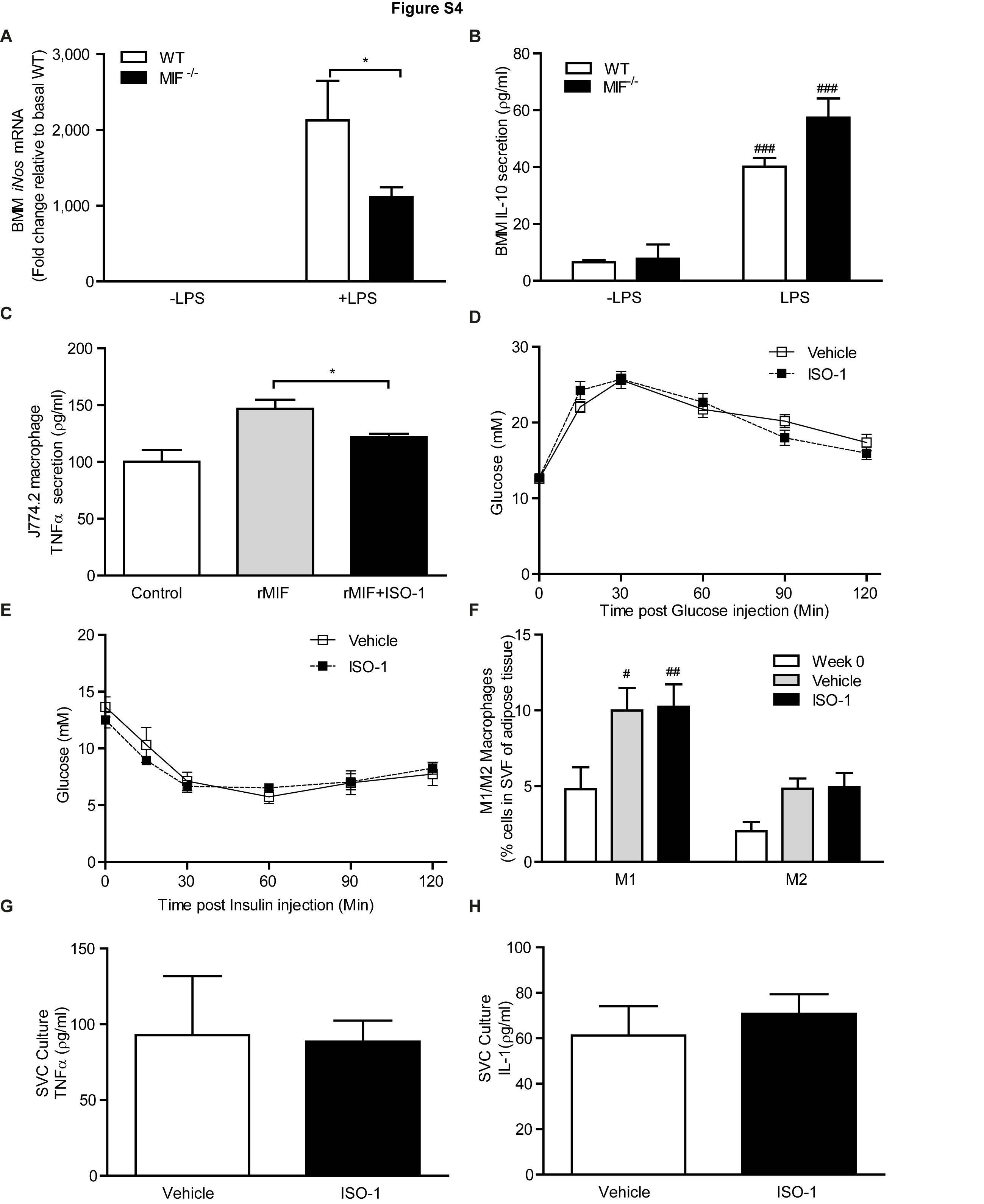

Supplement: Figure S5 — ISO-1 treatment inhibits MIF-induced TNFα cytokine secretion from J774.2 macrophages but cannot restore insulin sensitivity in vivo. (A) J774.2 macrophages were pre-treated with ISO-1 (50 µM/ml) for 1 hour prior to rMIF (100 ng/ml) stimulation for 3 hours. Media was harvested for TNFα cytokine secretion (n = 3, *p≤0.05, w.r.t MIF stimulated cells, # p≤0.05, w.r.t control cells. (B) GTT (1.5 g/kg glucose) in 4–6 hour fasted mice treated with or without ISO-1 (n = 10/group). (C) ITT (0.75 U/kg insulin) in 6 hour fasted mice treated with or without ISO-1 (n = 5/group). SVC harvested from mice treated with or without ISO-1 were cultured overnight. (A) TNFα and (E) IL-1β cytokine secretion into media was measured by ELISA (n = 4/group). (TIF) [file pone.0113369.s005.tif]
