# Supplementary material for: Macrophage Migration Inhibitory Factor Deficiency Ameliorates High-Fat Diet Induced Insulin Resistance in Mice with Reduced Adipose Inflammation and Hepatic Steatosis
Source: PLoS One. 2014 Nov 20;9(11):e113369. doi: 10.1371/journal.pone.0113369 (PMC4239060; doi:10.1371/journal.pone.0113369)
Supplement: File S1 — Supporting materials and methods. (DOC) [file pone.0113369.s006.doc]

**Supporting Information**

**Plasma metabolic analysis:** Fasting plasma cytokine levels were measured using multiplex ELISA (MesoScale Diagnostics, USA). Fasting plasma triacylglyceride, NEFA, cholesterol (WAKO, Diagnostics, USA) and alanine transaminase were quantified using commercial available kits (BioVision, Inc.CA). Fasting plasma leptin was measured using ultra-sensitive kits (R&D Systems, Europe,Ltd.) and MCP-1 using duo-set (R&D Systems Europe, Abingdon, United Kingdom).

**Hepatic triglyceride content:** Liver tissue was homogenized (50mg/ml) in RIPA buffer. Triacylglyceride levels were quantified using commercially available kit (WAKO diagnostics, USA).

**Histological Analyses:** Adipose and liver tissue was fixed in 10% formalin and paraffin embedded. Cross sections were prepared (5μM) using Leica EG1150H Machine. Haematoxylin and Eosin staining was conducted using Leica Autostainer XLabd and Leica CV530. Sections were analyzed using Nikon 80i transmission light microscope.

**RNA Isolation and QRT-PCR and Western Blot analysis**

RNA extracted from adipose and liver tissue (50mg/ml) using TRI Reagent® method as per manufacturer’s instructions. BMM and 3T3-L1-adipocytes (day10) were harvested in TRI Reagent® RNA (500ng-1000ng) was reverse transcribed using High Capacity cDNA archive kit (Applied Biosystems, Ireland). Expression of: *Tnfα, Il-1β, Glut-4, Irs-1, Cd36, Srebp-1c, Pparγ, Fasn, Dgat, Pgc-1α, Lpl, F4/80, Cd206* and *iNos* was measured by QRT-PCR using ABI Prism GeneAmp 7900HT Sequence Detection System (Applied Biosystems). Results were expressed in relative copy numbers after normalization against internal control GAPDH (Applied Biosystems). For western blot analysis adipose tissue (50mg), BMM and 3T3-L1-adipocytes were lysed in RIPA buffer. Protein concentration was determined by Bradford assay. Protein lysates (10-25μg) were heated-100°C for 5 minutes and applied to 4-20% polyacrylamide gradient gel and transferred to nitrocellulose membranes (Protan). Membranes were blocked in 10% skimmed milk in TBS-T (Marvel, Chivers Ireland Ltd.) for 1 hour. Blots were probed (1:1000 dilution) with primary antibodies to phosphorylated AKT, phosphorylated ERK, JNK, p38 and NFκB and β-actin over night at 4°C and detected using horseradish peroxidase conjugated anti-rabbit secondary antibody (1:1500-1:2000 dilution) for 90 minutes (Cell Signaling). Immunoreactive bands were visualized using an ECL reaction (Thermoscientific).

**ELISA**

Cytokine profile of media collected from adipose tissue, BMM, 3T3-L1-adipocytes and J774.2 macrophages was quantified by ELISA or multiplex mesoscale (R&D Systems Europe, Abingdon, United Kingdom).

**Conditioned Media Experiments**

J774.2 macrophages were grown in serum rich media in T75 tissue culture flasks at a density of 2X10^6^cells/ml. Media was collected after 24 hours from 1) unstimulated J774.2 macrophages, 2) J774.2 macrophages treated with rMIF (100ng/ml), 3) J774.2 macrophages treated with ISO-1 (50μM) (Merck, Ireland) and 4) J774.2 macrophages pretreated with ISO-1 (50μM) for 1 hour followed by rMIF (100ng/ml) treatment. Mature 3T3-L1 adipocytes were subsequently exposed to the collected conditioned media (CM) for 24 hours. Cells were subsequently lysed in RIPA buffer and insulin-stimulated ^3^H-glucose transport assay into adipocytes was carried out.

**Drug Intervention**

C57BL/6 WT male mice (aged 8-10 weeks) were fed HFD (45% kcal from palm oil) (Research Diets Inc., USA) or chow diet (10% kcal fat) (Harlan Teklad UK) *ab libitum* for 4 weeks. Mice were subsequently administered ISO-1 (10mg/Kg) (Merck, UK) by intraperitoneal (i.p.) injection once daily for 2 weeks. Separately, a subset of mice were administered ISO-1 (35mg/kg) by i.p. injection once daily for 3 days. Body weight was monitored weekly. Mice were fasted for 4-6 hours, anesthetized with isoflurane prior to cardiac puncture and were subsequently sacrificed by cervical dislocation. Plasma and tissue was harvested for further analysis.
